# Reducing Sedentary Time and Whole-Body Insulin Sensitivity in Metabolic Syndrome: A 6-Month Randomized Controlled Trial

**DOI:** 10.1249/MSS.0000000000003054

**Published:** 2022-10-13

**Authors:** TANJA SJÖROS, SAARA LAINE, TARU GARTHWAITE, HENRI VÄHÄ-YPYÄ, ELIISA LÖYTTYNIEMI, MIKKO KOIVUMÄKI, NOORA HOUTTU, Kirsi LAITINEN, Kari K. KALLIOKOSKI, HARRI SIEVÄNEN, TOMMI VASANKARI, JUHANI KNUUTI, ILKKA H.A. HEINONEN

**Affiliations:** 1 Turku PET Centre, University of Turku and Turku University Hospital, Turku, FINLAND; 2 The UKK Institute for Health Promotion Research, Tampere, FINLAND; 3 Department of Biostatistics, University of Turku, Turku, FINLAND; 4 Institute of Biomedicine, University of Turku, Turku, FINLAND; 5 Faculty of Medicine and Health Technology, Tampere University, Tampere, FINLAND; 6 Rydberg Laboratory of Applied Sciences, University of Halmstad, Halmstad, SWEDEN

**Keywords:** SEDENTARY BEHAVIOR, PHYSICAL ACTIVITY, METABOLIC SYNDROME, INSULIN RESISTANCE, OVERWEIGHT, ACCELEROMETRY

## Abstract

**Purpose:**

This study aimed to investigate whether a reduction in daily sedentary behavior (SB) improves insulin sensitivity in adults with metabolic syndrome in 6 months, without adding intentional exercise training.

**Methods:**

Sixty-four sedentary inactive middle-age adults with overweight and metabolic syndrome (mean (SD) age, 58 (7) yr; mean (SD) body mass index, 31.6 (4.3) kg·m^−2^; 27 men) were randomized into intervention and control groups. The 6-month individualized behavioral intervention supported by an interactive accelerometer and a mobile application aimed at reducing daily SB by 1 h compared with baseline. Insulin sensitivity by hyperinsulinemic euglycemic clamp, body composition by air displacement plethysmography, and fasting blood samples were analyzed before and after the intervention. SB and physical activity were measured with hip-worn accelerometers throughout the intervention.

**Results:**

SB decreased by 40 (95% confidence interval, 17–65) min·d^−1^, and moderate-to-vigorous physical activity increased by 20 (95% confidence interval, 11–28) min·d^−1^ on average in the intervention group with no significant changes in these outcomes in the control group. After 6 months, fasting plasma insulin decreased (~1 mU·L^−1^) in the intervention group compared with the control group (time–group, *P* = 0.0081), but insulin sensitivity did not change in either group. The changes in body mass or adiposity did not differ between groups. Among all participants, the changes in SB and body mass correlated inversely with the change in insulin sensitivity (*r* = −0.31, −0.44; *P* = 0.025, 0.0005, respectively).

**Conclusions:**

An intervention aimed at reducing daily SB resulted in slightly decreased fasting insulin, but had no effects on insulin sensitivity or body adiposity. However, as the change in insulin sensitivity associated with the changes in SB and body mass, multifaceted interventions targeting to weight loss are likely to be beneficial in improving whole-body insulin sensitivity.

The associations between measured sedentary behavior (SB) and metabolic disorders as well as premature death are well established (1–3). However, approximately 30–40 min of accelerometer-measured moderate-to-vigorous physical activity (MVPA) per day seems to wash out the detrimental effects of being sedentary (4). However, the health effects of standing, light physical activity (LPA), or nonexercise physical activity (PA) in everyday chores are far less certain. Despite the mounting epidemiological evidence, the physiological effects of reducing SB are not thoroughly understood (5,6). Behavioral interventions that aim to reduce SB may have small beneficial effects on common cardiometabolic risk markers, such as body fat percentage, waist circumference, and fasting insulin (7,8). However, in the aforementioned meta-analyses, some of the interventions included also exercise or dietary components in them. Therefore, the metabolic effects of reducing SB without adding exercise or altering diet remain inconclusive.

Metabolic syndrome (MetS) is a lifestyle-related cluster of metabolic disorders that are associated with a sedentary lifestyle and a positive energy balance and can lead to type 2 diabetes and cardiovascular diseases (9). Device-measured SB has been associated with several elements of MetS (10). However, these associations can be mediated by different behavior patterns and the amount of concomitant MVPA (11–13).

Insulin resistance is a gradually developing disorder and one of the early manifestations of type 2 diabetes, which can effectively be counteracted by exercise (14,15). Hyperinsulinemic euglycemic clamp (HEC) is considered the gold standard for measuring insulin sensitivity in humans (16). However, the long-term effects of reduced SB on HEC-measured insulin sensitivity have not previously been studied.

The amount of SB can be reduced by different behavioral strategies. Previously, counseling interventions have been able to reduce daily SB by 24–91 min·d^−1^ (17–19). In the short term (i.e., in acute crossover trials or in interventions lasting for less than a week), breaking up sitting with short activity breaks decreases postprandial plasma glucose and insulin levels (20–22). Moreover, replacing 5 h of daily sitting with standing and walking improved peripheral insulin sensitivity measured by a two-phase HEC in a 4-d crossover trial (23). In addition, replacing 1–2 h of daily SB with standing may slightly decrease body fat as well as fasting plasma glucose and insulin (24). However, there is still limited evidence about how LPA or standing as replacement to SB, without adding exercise or changing diet, can improve metabolic health (25).

A weakness in the previously reported long-term interventions (i.e., interventions lasting for more than 3 months) is that the PA and SB of the study participants have been measured with devices only for approximately 5–10 d before and at the end of the intervention, and not during the whole follow-up (26–29). It is therefore possible that the measurements have not detected some actual changes in behavior that have happened during the intervention.

The purpose of this randomized controlled trial was to investigate whether replacing 1 h of daily SB with standing or PA, without adding exercise, would improve whole-body insulin-stimulated glucose uptake (GU) measured by HEC, body composition, and MetS status in sedentary inactive adults with MetS during 6 months. Previously, we have reported that after 3 months, the increases in plasma insulin, insulin resistance index, and glycated hemoglobin (HbA_1c_) were attenuated in favor of the intervention (30). However, the primary outcome of GU was not measured after 3 months. Furthermore, we analyzed the participants’ accelerometer-measured SB and PA through the whole 6-month intervention to trace the actual behavior changes in the intervention (INT) and control (CONT) groups.

## MATERIALS AND METHODS

This study was a randomized controlled trial in free-living conditions. The study was conducted at the Turku PET Centre, Turku, Finland, between April 2017 and March 2020 according to good clinical practice and the Declaration of Helsinki. The participants gave their informed consent before entering the study. The study was approved by the Ethics Committee of the Hospital District of Southwestern Finland (16/1810/2017). The study is registered at ClinicalTrials.gov (NCT03101228, 05/04/2017).

### Study participants

The participants were recruited from the local community by newspaper advertisements and bulletin leaflets. The inclusion criteria for choosing the participants were as follows: age of 40–65 yr, body mass index (BMI) of 25–40 kg·m^−2^, fulfilling the criteria of MetS (31) (excluding diagnosed diabetes), SB time of at least 10 h·d^−1^ or 60% of accelerometer wear time in a 4-wk screening measurement, and self-reported physical inactivity (<150 min of moderate-intensity PA per week). The exclusion criteria were as follows: history of a cardiac event, diagnosed diabetes, abundant use of alcohol according to the national guidelines (12 units per week for women and 23 units per week for men), use of narcotics, smoking tobacco or consuming snuff tobacco, diagnosed depression or bipolar disorder, inability to understand written Finnish, and any chronic disease or condition that could create a hazard to the participant’s safety, endanger the study procedures, or interfere with the interpretation of study results.

### Anthropometric and metabolic measurements

HEC was performed as previously described by Sjöros et al. (15) and originally described by DeFronzo et al. (16). HEC was performed after at least 10 h of fasting. A primed-constant insulin (Actrapid, 100 U·mL^−1^; Novo Nordisk, Bagsvaerd, Denmark) infusion rate was 160 mU·min^−1^·m^−2^ of the participant’s body surface area during the first 4 min. From 4 to 7 min, the infusion rate was reduced to 80 mU·m^−2^·min^−1^, and from 7 min to the end of the clamp, it was kept constant at 40 mU·m^−2^·min^−1^. An exogenous 20% glucose infusion was started 4 min after the initiation of the insulin infusion, with a rate of milliliters per hour per participant’s body mass (kg) × 0.5; for example, for a person weighing 80 kg, the rate was 40 mL·h^−1^ ≈ 8 g of glucose per hour. At 10 min, the glucose infusion was doubled, and after that further adjusted according to blood glucose concentration to keep it as close as possible to the level of 5 mmol·L^−1^. Arterialized venous blood samples were collected every 5 min during the first 30 min and at steady state every 10 min to determine the glucose concentration for adjusting the glucose infusion rate. The whole-body insulin-stimulated GU rate was calculated from the measured steady-state glucose values and glucose infusion rate starting from 20 min after the start of the HEC. The outcome, M-value, represents whole-body GU as micromoles per kilogram of body mass per minute.

Venous blood samples were drawn after at least 10 h of fasting and analyzed at the Turku University Hospital Laboratory. Plasma insulin was determined by electrochemiluminescence immunoassay (Cobas 8000 e801; Roche Diagnostics GmbH, Mannheim, Germany). Plasma glucose was determined by enzymatic reference method with hexokinase GLUC3 and plasma triglycerides and HDL cholesterol by enzymatic colorimetric tests (Cobas 8000 c702; Roche Diagnostics GmbH). HbA_1c_ was determined by turbidimetric inhibition immunoassay (Cobas 6000 c501; Roche Diagnostics GmbH). Homeostasis model for insulin resistance index (HOMA-IR) was calculated with formula glucose × insulin/22.5.

Body mass, body fat, and fat-free mass (FFM) were measured by air displacement plethysmography (Cosmed USA, Concord, CA) after at least 4 h of fasting. Body height was measured with a wall-mounted stadiometer. Waist circumference in 0.1 cm was measured with a flexible measuring tape midline between the iliac crest and the lowest rib, repeated twice or until the same measure was obtained twice. One researcher did all the waist circumference measurements. Blood pressure was measured with a digital blood pressure monitor (Apteq AE701f; Rossmax International Ltd, Taipei, Taiwan) in a seated position after at least 5 min of sitting. The mean of two to three measurements was used as the outcome measure. MetS score was calculated as the sum of *z* scores of the following outcomes: waist circumference, mean blood pressure, fasting plasma glucose, insulin, and HDL/triglyceride ratio (32).

### Accelerometry

SB and PA were measured during waking hours through the whole intervention with a hip-worn triaxial accelerometer (Movesense; Suunto, Vantaa, Finland) with embedded measurement and analysis algorithms (ExSed; UKK Institute, Tampere, Finland). The baseline SB and PA were measured for 4 wk at the screening phase with a hip-worn triaxial accelerometer (UKKAM30; UKK-institute, Tampere, Finland). The collected accelerometer data were analyzed in 6-s epochs using validated mean amplitude deviation (MAD) and angle for posture estimation (APE) methods. The epoch-wise MAD values were converted to metabolic equivalents (METs) (3.5 mL·kg^−1^·min^−1^ of oxygen consumption) (33). LPA was defined as 1.5 < 3.0 MET, MVPA as ≥3.0 MET, and vigorous PA (VPA) as ≥6.0 MET. The amount of VPA accumulated during the study was marginal (median, 0.2 min·d^−1^ during screening and 0.6 min·d^−1^ during intervention), the distribution was skewed, and thus, the change in VPA could not be reliably analyzed. Therefore, VPA was added to moderate-intensity PA and presented as MVPA.

The body posture was determined with the APE method only for the epochs, which had a MET value lower than 1.5 (34). During walking, the accelerometer orientation in terms of the gravity vector was taken as the reference vector. The posture was determined from the incident accelerometer orientation in relation to the reference vector. The epochs having APE values less than 11.6° were classified as standing and epochs having APE value at least 11.6° as SB. In free-living conditions, the agreement between the posture classification from simultaneous thigh-worn and hip-worn data has been about 90% (34). The embedded ExSed algorithms are virtually identical to the MAD and APE algorithms.

The step detection algorithm splits the measured acceleration into vertical and horizontal components. The vertical component is band-pass filtered (1–4 Hz), and positive values are integrated. When the integral value exceeds the specified limit, a step is detected (34).

A period was classified as nonwear time, if the acceleration of each three measurement axes remained within 187.5-m*g* range at least for 30 min. Wear time of 10–19 h·d^−1^ was considered valid. Daily measurement time exceeding 19 h indicates that a participant has likely worn the accelerometer while sleeping, and therefore, measurement time exceeding 19 h·d^−1^ was subtracted from the SB time. In addition to time spent in each behavior, proportions of different activity intensities per day were calculated as percentage of wear time. In addition, to estimate the fluctuations over time in measured SB and PA, the duration of the intervention period was split into quartiles, and the daily mean SB, standing, LPA, MVPA, steps, and breaks in SB during each quartile were calculated.

### Intervention

After the baseline measurements, the participants were randomly allocated by a statistician to the INT and CONT groups using random permuted blocks with 1:1 allocation ratio and block size of 44, performed separately for men and women.

The participants in the INT group received a 1-h tailored personal counseling session with a physiotherapist, where they were instructed to reduce their SB by 1 h·d^−1^ compared with baseline. The means to achieve this goal were planned individually according to each participant’s preferences and supported by a mobile application (www.exsed.com, ExSed; UKK Institute, Tampere, Finland) connected to the accelerometer worn on the hip during waking hours of the whole intervention. The counseling session included tips for different behaviors as well as strategies for goal setting and getting support. The application provided a timely visual summary of the measured SB, standing, LPA, MVPA, and steps, thus enabling continuous self-monitoring by the participants. The target levels of daily SB and PA were set according to the baseline measurements by reducing 1 h of SB and adding an equivalent time of standing, LPA, and MVPA, divided evenly or according to each participant’s preferences. However, a maximum of 20 min was added to MVPA. During the intervention, the participants received two to three phone calls from the instructor and visited the research center at least once to gain support in achieving the goals and to assure the functioning of the devises.

The CONT participants were instructed to maintain their habitual PA and SB during the intervention and received the mobile application and accelerometer. The goals for SB and PA in the application were set according to the baseline accelerometer measurements. Both groups were instructed not to alter their diet during the intervention, and this was assessed by 4-d food diaries before and at the end of the intervention. The mean daily energy intake was calculated with computerized software (AivoDiet 2.2.0.1; Aivo, Turku, Finland). The duration of the intervention was 5–6 months, after which all measurements done at the baseline were repeated.

### Statistical methods

The sample size was determined according to the following power calculations: Based on the earlier finding that GU was increased by 2.4 μmol·kg^−1^·min^−1^ after 2 wk of moderate-intensity exercise (35), we estimated that reduced SB intervention would increase GU by 1.9 (SD, 1.8) μmol·kg^−1^·min^−1^, which represents a 6% change from the baseline. It was estimated that in the CONT group, GU would increase by 0.2 μmol·kg^−1^·min^−1^. To detect a statistically significant change during intervention and compared with the CONT group, we calculated that 24 participants were needed in both groups (*α* = 0.05, 1 − *β* = 0.9). To allow for possible dropouts and technical problems in the measurements, 64 subjects were recruited. The differences between groups at the baseline were tested with Student’s *t*-test or Fisher’s exact test when applicable. The changes over time and across groups were tested by linear mixed models for repeated measurements with three categorical variables (time (within-factor), group, sex) and the interaction term (group–time). Pairwise comparisons were adjusted with Tukey–Kramer adjustment for multiple comparisons. When evaluating the changes in measured SB and PA time, mean daily accelerometer wear time was included as a covariate in the model. The normal distributions of the residuals were evaluated visually, and logarithmic (log10) transformations were performed when necessary to fulfill normal distribution assumption of the residuals. The associations between changes in the measured outcomes were tested with Pearson correlation coefficient. If not otherwise stated, data are expressed as means (SD) or model-based means (95% confidence intervals) when applicable. In case of a skewed distribution, medians with first and third quartiles (Q1, Q3) are presented. The level of statistical significance was set at 5% (two-tailed). The correlation analyses were carried out with IBM SPSS Statistics 27.0 (IBM Corp., Armonk, NY) and all the other analyses with the SAS 9.4 and JMP pro 15 for Windows (SAS Institute Inc., Cary, NC).

### Additional analyses

We ran additional analyses by dividing the participants into two groups according to the changes in measured SB as a proportion of the daily wear time of the accelerometer. The participants who reduced their daily SB by at least three percentage points compared with the baseline (that equals about a 27-min reduction in SB with 15-h wear time) were defined as “more active” (*n* = 30), and the participants who either increased their SB or reduced it less than three percentage points compared with the baseline were defined as “continuously sedentary” (*n* = 26). This cut-point was chosen because it created relatively even number of participants in each group, and the assumption of normal distribution of the residuals in the statistical model was fulfilled. The participants with missing accelerometer data during the intervention (*n* = 8) were allocated according to the original randomization, resulting in 34 participants (with 26 from the intervention and 8 from the control group) in the more active and 30 (with 7 from the intervention and 23 from the control group) in the continuously sedentary group.

## RESULTS

In total, 263 individuals volunteered, of which 155 participated in the screening measurements and 64 participants were included (Fig. S1, Supplemental Digital Content, Study flow diagram, http://links.lww.com/MSS/C722). One participant in the INT group discontinued intervention because of personal reasons. Three participants in the CONT group discontinued intervention, two because of personal reasons and one because of low back pain. The baseline characteristics of the INT and CONT groups are presented in Table 1.

**TABLE 1:** Study participant characteristics at the baseline.

Group	Intervention (INT)	Control (CONT)
*n*	33	31
Men, *n* (%)	13 (39)	14 (45)
Age, yr	59 (6)	57 (8)
Antihypertensive medication, *n* (%)	17 (52)	17 (55)
Cholesterol medication, *n* (%)	9 (27)	5 (16)
Energy intake, kJ·d^−1^	7271 (1603)	7789 (1725)
Energy intake/body mass, kJ·kg^−1^·d^−1^	79.7 (16.1)	84.8 (22.1)
Metabolic variables		
MetS score	0.71 (3.01)	−0.42 (3.15)
SBP, mm Hg	146 (15)	139 (16)
DBP, mm Hg	89 (8)	88 (9)
HR, bpm	69 (10)	66 (6)
Waist circumference, cm	111.1 (11.6)	110.7 (11.1)
Body height, cm	170.9 (8.7)	172.1 (8.4)
Body mass, kg	92.4 (16.6)	94.1 (15.8)
BMI, kg·m^−2^	31.5 (4.0)	31.7 (4.6)
Body fat, %	43.1 (8.0)	43.1 (8.0)
FFM, kg	52.6 (11.9)	53.2 (9.8)
Median M-value, μmol·kg^−1^·min^−1^ (Q1, Q3)	15.3 (10.7, 21.0)	13.9 (9.8, 21.0)
fP-Glucose, mmol·L^−1^	5.9 (0.5)	5.8 (0.4)
Median fP-Insulin, mU·L^−1^ (Q1, Q3)	9 (7, 13)	11 (7, 17)
Median HOMA-IR (Q1, Q3)	2.4 (1.8, 3.8)	2.8 (1.6, 4.3)
HbA_1c_, mmol·L^−1^	37 (2.8)	36.3 (2.7)
Accelerometer variables		
Accelerometry, d	25.8 (3.7)	25.7 (3.4)
Accelerometry, h·d^−1^	14.5 (1.0)	14.6 (1.0)
Sedentary time, h·d^−1^	10.0 (0.9)	10.1 (1.1)
Sedentary proportion, %·d^−1^	69.3 (5.6)	68.8 (6.6)
Standing time, h·d^−1^	1.8 (0.6)	1.8 (0.6)
LPA, h·d^−1^	1.7 (0.4)	1.8 (0.5)
MVPA, h·d^−1^	0.96 (0.31)	0.97 (0.34)
Breaks in sedentary time, n per day	28 (8)	29 (8)
Steps, n per day	5204 (1910)	5091 (1760)

Unless otherwise stated, the results are presented as mean (SD). The differences between groups were tested with *t*-tests or Fisher’s exact test when applicable, and no significant differences between groups were found.

DBP, diastolic blood pressure; fP-Glucose, fasting plasma glucose; fP-Insulin, fasting plasma insulin; HR, resting heart rate; MetS score, sum score of waist circumference, mean blood pressure, fasting plasma glucose, insulin, and the HDL/triglyceride ratio; M-value, whole-body GU in HEC; Q1, first quartile; Q3, third quartile; SBP, systolic blood pressure.

### Accelerometry

**FIGURE 1:**
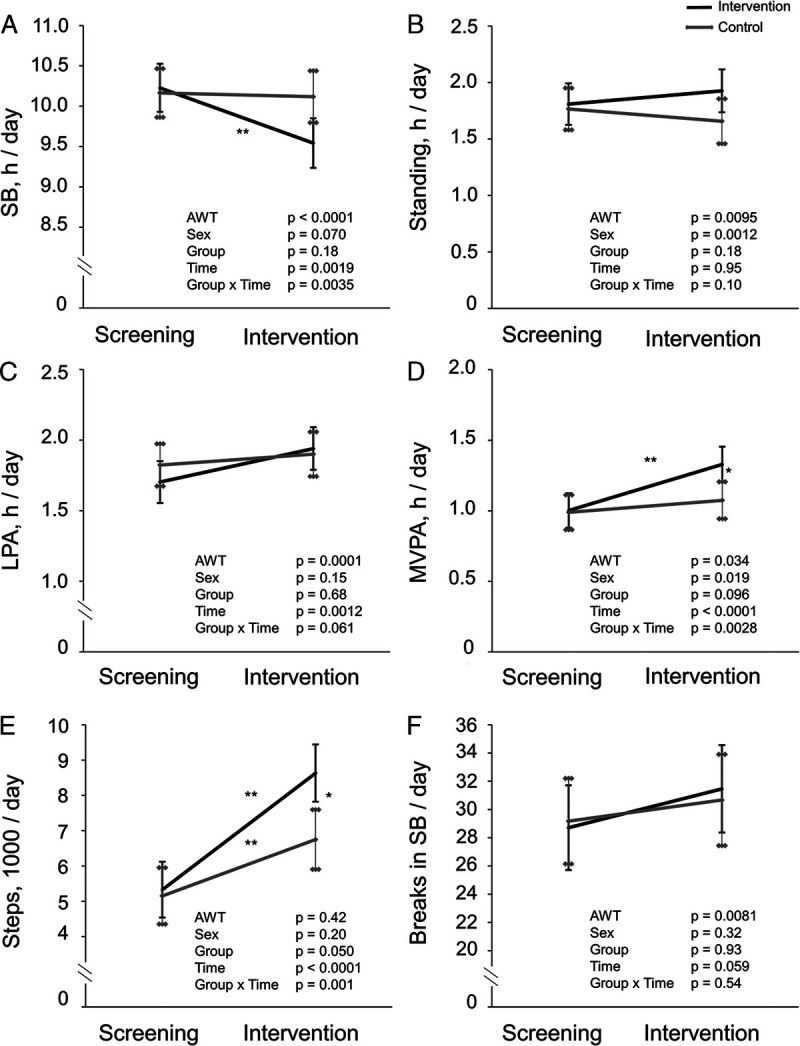
Accelerometer-measured PA and SB of the intervention (*black line*) and control (*gray line*) groups during a 4-wk (mean (SD), 26 (4) d of accelerometer data collection) screening phase (Screening) and a 6-month (median (Q1, Q3), 117 (74, 142) d of accelerometer data collection) intervention phase (Intervention) presented as model-based means with 95% confidence intervals. Accelerometer wear time and sex were included as covariables in all the analyses. A, SB time per day. B, Standing time per day. C, LPA per day. D, MVPA per day. E, Steps per day. F, Breaks in SB per day. AWT, accelerometer wear time; within- or between-group difference, *P < 0.05; **P < 0.01.

The mean (SD) duration of the intervention was 171 (36) d. Accelerometer data of 56 participants were successfully collected during the intervention with a median (Q1, Q3) duration of 117 (74, 142) d. The data collection of eight participants failed, one because of discontinued participation in the study and seven because of technical errors. During the intervention, SB decreased by approximately 40 min·d^−1^ (5% of the wear time of the accelerometer) compared with baseline in the INT group, whereas no change was detected in the CONT group (Fig. 1). Standing time did not change in either group during the intervention. LPA increased on average by 10 min·d^−1^ during the intervention, but the difference between groups was not significant.

MVPA increased in the INT group by 20 min·d^−1^ on average, whereas in the CONT group, the change was not significant. Daily steps increased on average by 3300 steps in the INT group and by 1600 steps in the CONT group (Fig. 1). Sex was a significant factor in the models estimating standing and MVPA, women spent more time standing, whereas men had more MVPA (data not shown).

**FIGURE 2:**
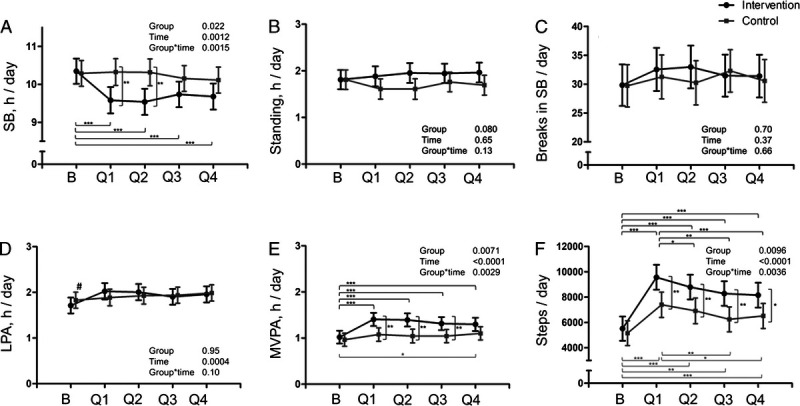
Accelerometer-measured PA and SB of the intervention (*black dots*) and control (*gray squares*) groups during a 4-wk screening phase (Baseline; with mean (SD) of 26 (4) d of accelerometer data collection) and a 6-month intervention phase in quartiles (Q1–Q4; with means (SD) of 30 (10), 29 (11), 33 (12), and 32 (12) d of accelerometer data collection, respectively) presented as model-based means with 95% confidence intervals. Accelerometer wear time and sex were included as covariables in all the analyses. A, SB time per day. B, Standing time per day. C, Breaks in SB per day. D, LPA per day. E, MVPA per day. F, Steps per day. B, baseline; Q1, first quartile of the intervention; Q2, second quartile of the intervention; Q3, third quartile of the intervention; Q4, fourth quartile of the intervention. Within- or between-group difference significant at the level of *P < 0.05, **P < 0.01, ***P < 0.001; #whole-group baseline mean significantly different from all quartiles Q1–Q4 at the level of *P* < 0.01.

The duration of the intervention period was split into quartiles, and data collection succeeded as follows: first quartile, *n* = 46 (mean (SD), 30 (10) d); second quartile, *n* = 46 (mean (SD), 29 (11) d); third quartile, *n* = 52 (mean (SD), 33 (12) d); and fourth quartile, *n* = 49 (mean (SD), 32 (12) d). SB decreased and MVPA increased significantly between the baseline and all four quartiles of the intervention in the INT group (Fig. 2). However, the CONT group also increased MVPA during the last quartile. Step count increased significantly between baseline and all four quartiles of the intervention in both groups but also decreased significantly after the first quartile of the intervention (Fig. 2). However, the difference in the step count of the INT and CONT groups remained significant throughout the four quartiles of the intervention (Fig. 2).

### Anthropometric and metabolic outcomes

**FIGURE 3:**
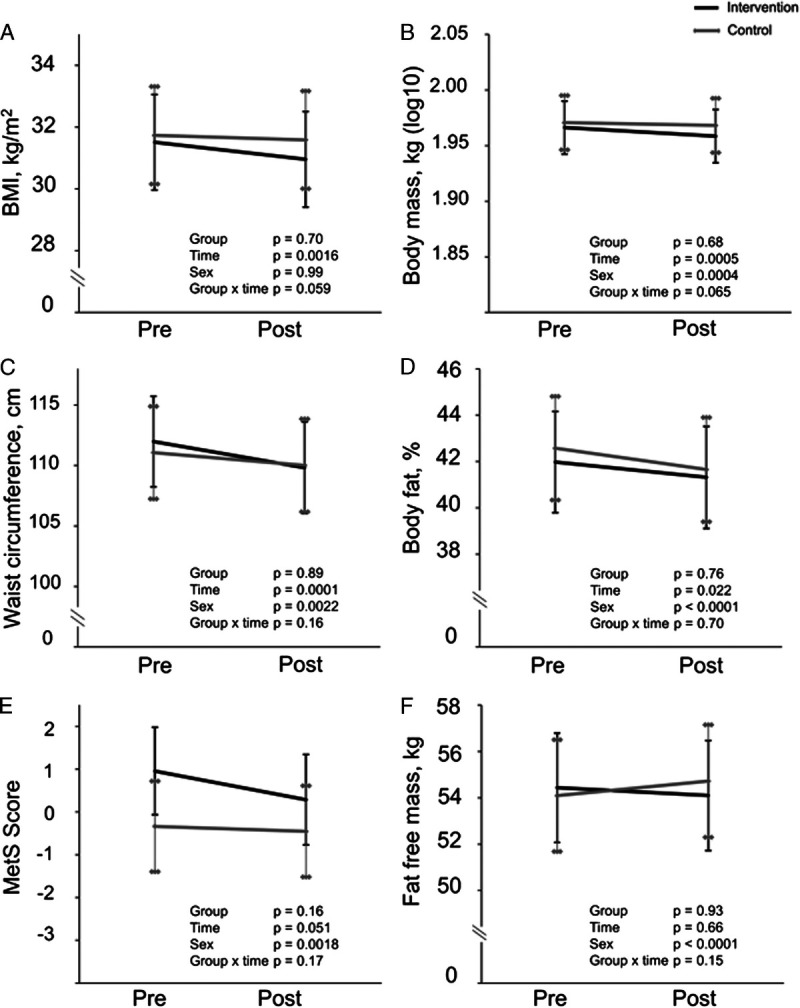
Anthropometric results of the intervention (*black line*) and control (*gray line*) groups before and after the intervention presented as model-based means with 95% confidence intervals. Sex was included as a covariable in all the analyses. A, BMI. B, Body mass. C, Waist circumference. D, Body fat percentage measured by air displacement plethysmography. E, MetS score, sum score of waist circumference, mean blood pressure, fasting plasma glucose, insulin, and HDL/triglyceride ratio. F, FFM measured by air displacement plethysmography.

**FIGURE 4:**
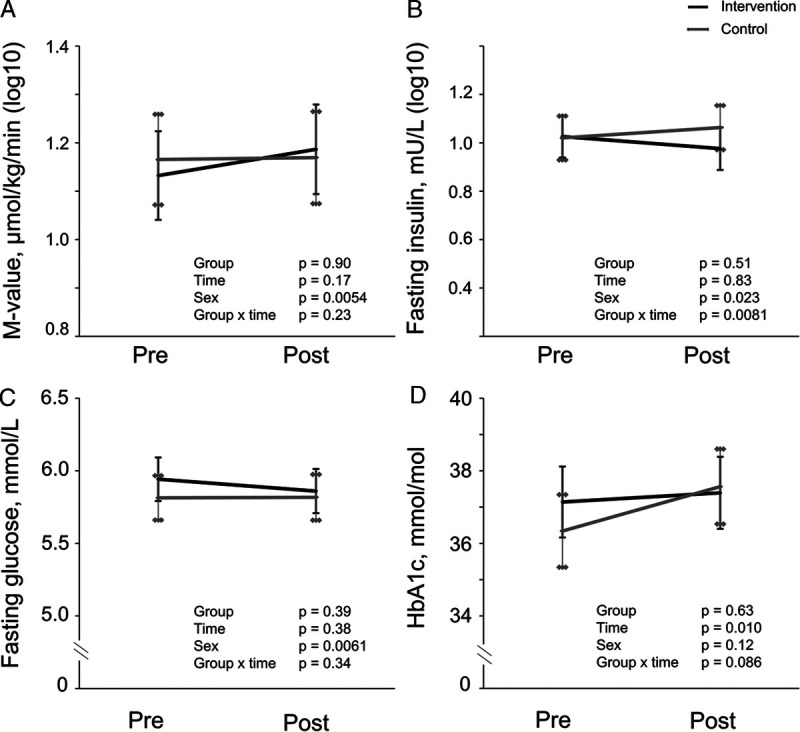
Metabolic results of the intervention (*black line*) and control (*gray line*) groups before and after the intervention presented as model-based means with 95% confidence intervals. Sex was included as a covariable in all the analyses. A, M-value, whole-body insulin-stimulated GU in HEC. B, Fasting plasma insulin. C, Fasting plasma glucose. D, HbA_1c_.

**FIGURE 5:**
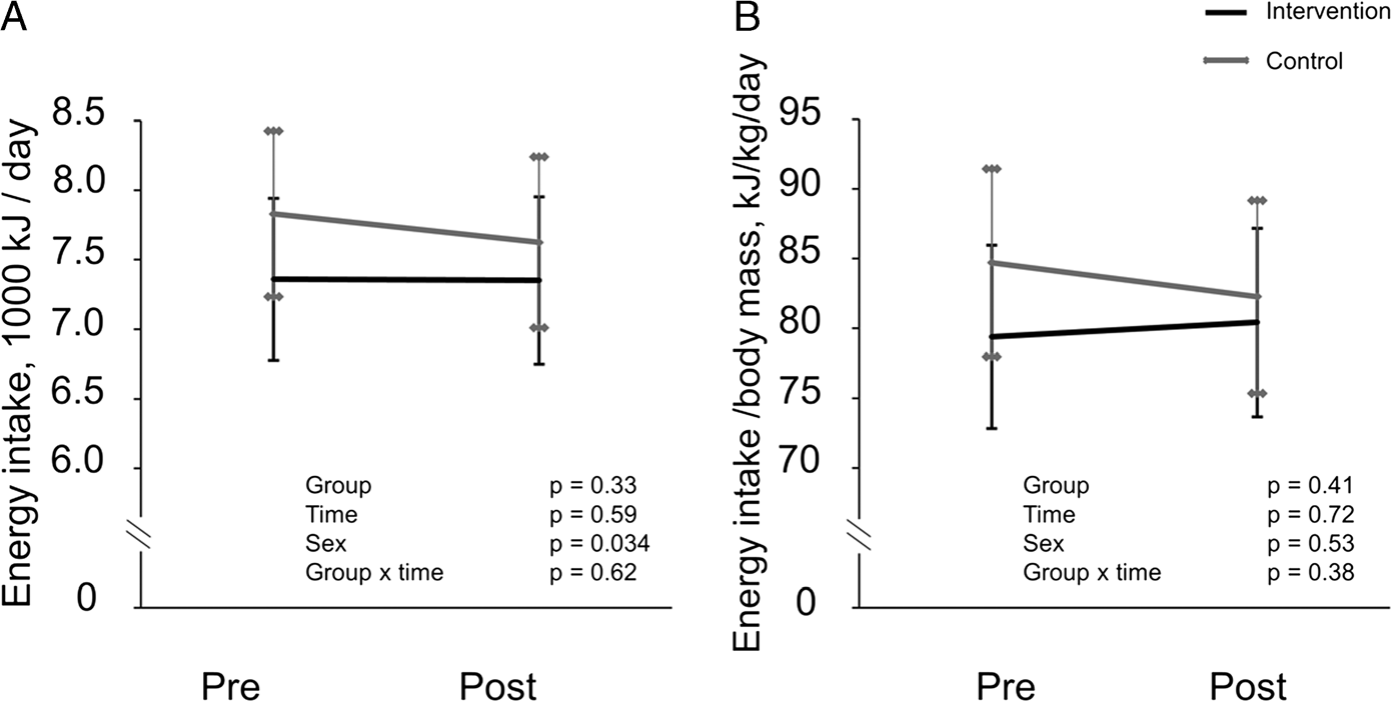
Energy intake of the intervention (*black line*) and control (*gray line*) groups calculated from 4-d food diaries (including one weekend day) before and at the end of the intervention presented as model-based means with 95% confidence intervals. Sex was included as a covariable in all the analyses. A, Energy intake per day. B, Energy intake per body mass per day.

Body mass, BMI, and waist circumference decreased similarly in both groups (Fig. 3), with no changes in insulin sensitivity (M-value in HEC), plasma glucose, FFM, or the MetS score (Figs. 3, 4). The change in body mass was ~−0.5 kg on average. HbA_1c_ increased during the intervention with no difference between groups (Fig. 4). Fasting insulin decreased in the INT group compared with the CONT group (Fig. 4), as did HOMA-IR (group–time, *P* = 0.009). The mean change difference between groups in fasting insulin was ~1 mU·L^−1^. Energy intake did not change during the intervention in either group (Fig. 5). The insulin and glucose values during HEC are presented in Table S1 (Supplemental Digital Content, http://links.lww.com/MSS/C722).

The change in insulin sensitivity was inversely associated with the changes in MetS score, BMI, body mass, fasting glucose, and SB percentage (Table 2). The changes in daily steps and MVPA were inversely correlated with the changes in waist circumference and HbA_1c_, and the change in LPA was inversely correlated with changes in BMI, body mass, and FFM. In addition, the change in energy intake was positively associated with the changes in BMI, body mass, and fasting glucose (Table 2).

**TABLE 2:** Pearson correlation coefficients between the changes in different metabolic, cardiovascular, and PA markers (post–pre Δ values) during the intervention.

	Δ MetS score	Δ WC, cm	Δ BMI, kg·m^−2^	Δ Body Mass, kg	Δ Body Fat %	Δ FFM, kg	Δ M-Value	Δ fInsulin, mU·L^−1^	Δ fGlucose, mmol·L^−1^	Δ HbA_1c_, mmol·L^−1^	Δ Energy Intake, kJ	Δ Energy Intake, kJ·kg^−1^	Δ SB %	Δ Standing %	Δ LPA %	Δ MVPA %	Δ Steps per Day	Δ Breaks in SB per Day
Δ MetS score	1	0.32*	0.35*	0.36**	−0.06	0.24	−0.30*	0.51**	0.55**	0.10	0.21	0.15	−0.03	0.17	−0.09	−0.12	−0.15	−0.02
Δ WC, cm		1	0.53**	0.54**	0.13	0.16	−0.23	0.20	0.10	0.24	0.25	0.19	0.09	0.18	−0.25	−0.29*	−0.30*	−0.03
Δ BMI, kg·m^−2^			1	1.00**	0.22	0.30*	−0.45**	0.21	0.23	0.31*	0.32*	0.19	0.27	−0.10	−0.37**	−0.19	−0.23	−0.19
Δ Body mass, kg				1	0.22	0.30*	−0.44**	0.23	0.23	0.32*	0.33*	0.19	0.27	−0.11	−0.37**	−0.17	−0.22	−0.18
Δ Body fat %					1	−0.85**	−0.03	−0.02	0.01	0.15	0.01	0.00	0.12	−0.21	0.09	−0.07	−0.12	−0.22
Δ FFM, kg						1	−0.21	0.18	0.14	−0.01	0.19	0.13	0.01	0.17	−0.28*	−0.04	−0.01	0.08
Δ M-value							1	−0.22	−0.32*	−0.11	−0.19	−0.13	−0.31*	0.24	0.26	0.17	0.19	0.02
Δ fInsulin, mU·L^−1^								1	0.23	0.18	0.15	0.10	0.17	−0.22	−0.07	−0.02	−0.09	−0.08
Δ fGlucose, mmol·L^−1^									1	0.11	0.49**	0.51**	0.00	0.13	−0.16	−0.03	−0.17	−0.27
Δ HbA_1c_, mmol·L^−1^										1	0.01	−0.02	0.16	0.00	−0.14	−0.28*	−0.32*	−0.17
Δ Energy intake, kJ											1	0.97**	−0.08	0.12	0.05	−0.03	−0.13	−0.09
Δ Energy intake, kJ·kg^−1^												1	−0.13	0.17	0.05	0.01	−0.10	−0.08
Δ SB %													1	−0.79***	−0.69**	−0.69**	−0.55**	−0.38**
Δ Standing %														1	0.24	0.25	0.20	0.17
Δ LPA %															1	0.46**	0.37**	0.35**
Δ MVPA %																1	0.79**	0.39**
Δ Steps per day																	1	0.48**
Δ Breaks in SB per day																		1

Δ, the change from preintervention to postintervention measurements in metabolic and cardiovascular outcomes, and from screening to intervention in accelerometry outcomes; fInsulin, fasting plasma insulin, fGlucose, fasting plasma glucose; LPA, LPA measured by accelerometry; MetS score, MetS severity score (sum of waist circumference, mean blood pressure, fasting plasma glucose, insulin, and HDL/triglyceride-ratio); M-value, whole-body insulin-stimulated GU measured by HEC; MVPA, MVPA measured by accelerometry; SB, SB measured by accelerometry; WC, waist circumference.

*Significant at the level of *P* < 0.05.

**Significant at the level of *P* < 0.01.

### Additional analyses

**FIGURE 6:**
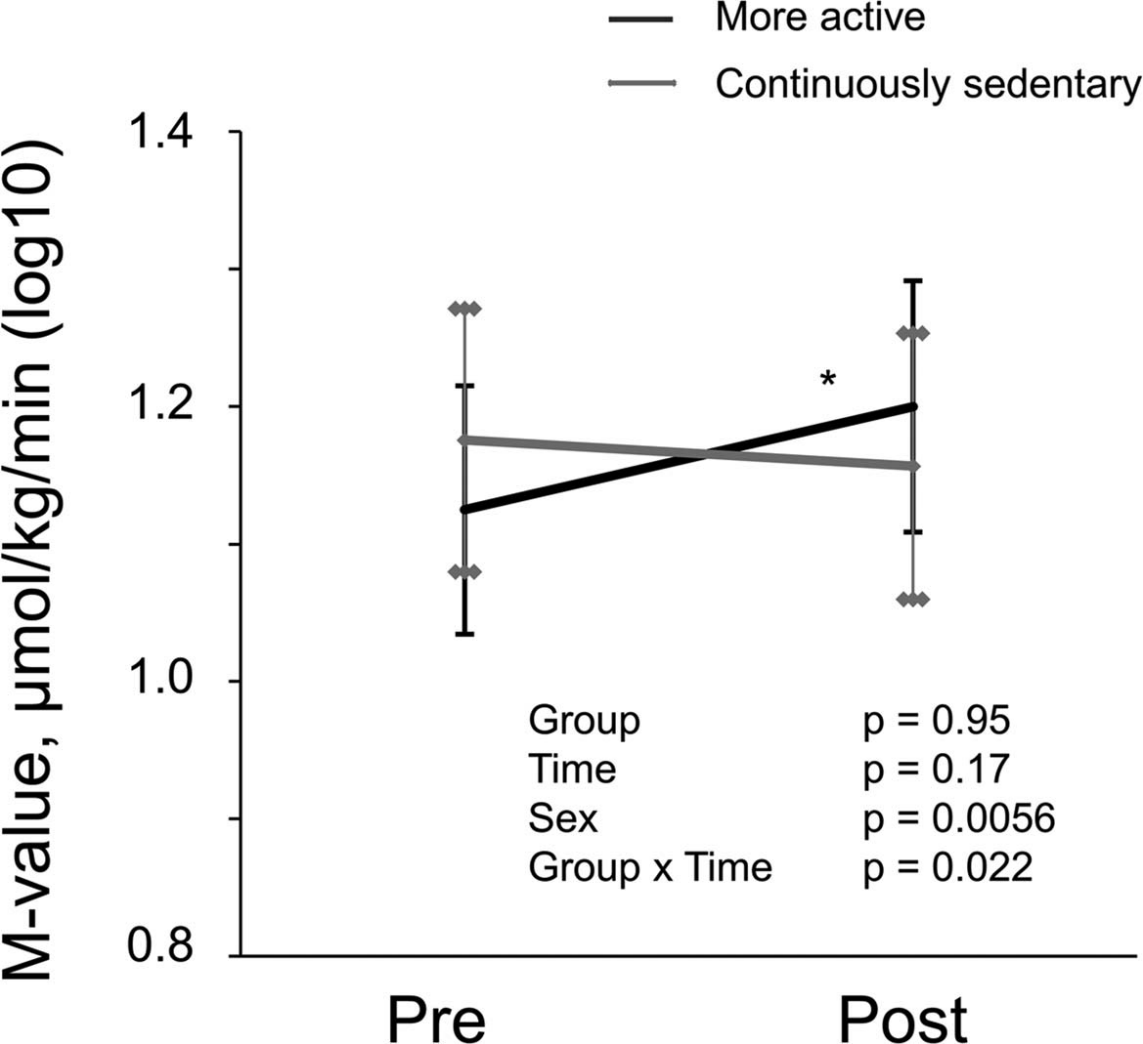
Whole-body insulin-stimulated GU (M-value) measured by HEC in more active (accelerometer-measured SB decreased by at least 3 percentage points during intervention compared with screening, *black line*) and continuously sedentary (accelerometer-measured SB increased, or decreased less than 3 percentage points during intervention compared with screening, *gray line*) participants before and after the intervention presented as model-based means with 95% confidence intervals. Sex was included as a covariable in the analysis.

When the participants were divided into two groups according to the changes in accelerometer-measured SB, insulin sensitivity increased in the more active group compared with the continuously sedentary group (Fig. 6). GU increased among the more active by 1 μmol·kg^−1^·min^−1^ (7.5%) on average. The numerical estimates (model-based means (SE)) of the results presented in Figures 1, 3, 4, 5, and 6 are presented in Table S2 (Supplemental Digital Content, http://links.lww.com/MSS/C722).

## DISCUSSION

In this study, a tailored intervention aimed to reduce SB by 1 h·d^−1^ resulted in a 40-min decrease in daily SB and a concomitant 20-min increase in MVPA during 6 months, with no significant changes in standing or LPA time. Moreover, we discovered a difference between the INT and CONT groups in the change in fasting insulin after 6 months. The whole-body insulin sensitivity measured by HEC did not change. Nevertheless, a trend toward beneficial changes in the INT group was consistent across several study parameters, and the additional analyses suggest that an actual reduction in measured SB also increases whole-body insulin sensitivity. However, based on our results, it seems that aiming at a 1-h reduction in daily SB by replacing it with nonexercise PA is not sufficient to induce major changes in metabolic health.

### Insulin resistance

Insulin resistance is currently considered a protective mechanism against hyperglycemia-induced hyperinsulinemia in plasma and subsequent hyperglycemia-induced tissue damage (36). Therefore, plasma insulin and glucose levels should decrease before any improvements in insulin sensitivity. Otherwise, enhanced insulin sensitivity would increase glucolipotoxicity in the tissues. In that perspective, the minor decrease in fasting insulin in the INT group of this study can be seen as the first step toward improved glucose metabolism and insulin sensitivity. Correspondingly, 3 wk of interrupting sitting improved fasting glucose but not glucose tolerance (37). However, aerobic and resistance exercises, which consist of MVPA, can increase insulin sensitivity very rapidly and even if performed in short bouts (38). Possibly more VPA would be needed to induce a significant improvement in insulin sensitivity in this study. Interestingly, in an earlier 6-month intervention study with sedentary adults, fasting insulin and waist circumference decreased with only 13-min increase in measured standing time (27). In addition, belonging to the intervention group seemed to protect against rising blood glucose levels at 3 months, whether or not a behavior change was detected by accelerometers, but in 12 months, this difference between intervention and control groups disappeared (28,39). Similarly, in our study, after 3 months HbA_1c_ increased in the CONT group compared with the INT group (30), but at 6 months, this difference was diluted, whereas the difference in plasma insulin remained significant. However, in a large cluster-randomized study, a small decrease in fasting plasma glucose was detected after 12 months as a result of replacing 45 min·d^−1^ of occupational sitting with standing (40). However, it should be noted that both accelerometry and statistical methods in the aforementioned studies differed from the methods used in this study. Therefore, direct comparisons considering the amounts of measured SB or PA cannot be made.

### Behavior change

This intervention was successful in reducing SB, but the mean change during the intervention (40 min·d^−1^) was less than the target level of 1 h. In addition, MVPA and step count increased significantly more in the INT group compared with the CONT group. When looking at the behavior changes in quartiles of the intervention period, the decrease in SB was more pronounced during the first two quartiles (Fig. 2). This is supported by previous studies where the intended change in SB has been more pronounced in the beginning of the intervention (29). The increase in the step count (that reflects well the overall PA) started to decline already after the first quartile (Fig. 2). However, the between-group difference in the step count favoring the INT group remained significant throughout the intervention. Interestingly, during the last quartile, the CONT group also increased MVPA, which may partly have affected the study outcomes.

The intervention aimed at replacing SB with standing and nonexercise PA, but during the whole 6-month intervention, the participants were able to sustain only the increase in MVPA (consisting mainly of moderate-intensity PA), whereas during the first 3 months, the mean durations of LPA and standing also increased, as previously reported (30). It may be easier to add moderate-intensity PA to the daily activities instead of altering daily sedentary chores to lightly active ones. For example, many of the participants in this study related they saw walking as the easiest way to increase daily PA. Measured by accelerometers, walking is most often classified as MVPA. Moreover, social and physical environments are important factors influencing individual PA, and therefore, workplace interventions may be more effective in increasing daily standing and LPA (29). Individual counseling may have more potential in increasing PA during free time, and MVPA and exercise are possibly the most feasible means to do that.

### Effectiveness of replacing SB with PA in daily activities

Even if the amount of MVPA and step count significantly increased, the intervention was unable to enhance whole-body insulin sensitivity measured by HEC. The (nearly total) lack of VPA may be the reason that MVPA was not effective in improving insulin sensitivity in this study. It is possible that VPA rather than moderate PA is needed to gain health benefits in adults with overweight (41,42). Alternatively, longer bouts of MVPA might have been needed. The amount of measured MVPA is dependent on the analysis method used and possible data smoothing (43). In this study, MVPA was measured in 6-s intervals, and thus, the daily increase (20 min in INT) may consist of very short bouts. Even if the length of MVPA bouts is no longer considered essential in gaining health benefits (44), it is still possible that PA bout duration plays a role in the health promotion of people with overweight and low cardiorespiratory fitness (CRF). Estimated by personal cut-points, people with low CRF gained more MVPA in their daily chores than people with high CRF; but only long bouts of VPA and MVPA were positively associated with CRF (43). At the baseline, the participants of the current study were unfit (average V̇O_2peak_ was 21 mL·kg^−1^·min^−1^ in women and 26 mL·kg^−1^·min^−1^ in men) (45). Possibly people with low CRF would need longer bouts of MVPA to gain significant health benefits because they may be unable to further increase the intensity of their daily PA, but whether or not this applies also to insulin sensitivity remains unresolved.

### Methodological consideration

In some studies, metabolic markers or physical functioning have modestly improved even if no changes in device-measured SB were detected at the end of the intervention (27,46). It is, however, possible that the intervention groups reduced their SB at some point of the intervention, but the measurements were unable to detect this altered behavior. Therefore, it can be reasoned that SB and PA should be measured by validated devices during the whole intervention, to detect the actual changes in behavior during the intervention, as was done in the present study.

### Strengths and limitations

Key strengths of this study are the randomized controlled trial design, gold standard method for measuring whole-body insulin sensitivity, and the 6-month assessment of SB and PA by accelerometry. A limitation was that because of the nature of the intervention, blinding of the participants was not possible. Moreover, the food diaries were collected only twice, during 4 consecutive days (including one weekend day) before and at the end of the intervention. Detailed instructions were given and the diaries were checked with a portion picture booklet during a study visit to assure reliable reporting, but there is a risk for underreporting or altered dietary habits during data collection. According to the food diaries, energy intake did not change significantly in either group. However, the change in energy intake was correlated to the changes in body mass, BMI, and fasting plasma glucose. This may indicate that some participants (who either did or did not change their SB) possibly changed their diet, and this could have led to a weight loss or gain during the intervention, and also contributed to the plasma glucose levels.

### Clinical implications

Insulin sensitivity is a multifactorial phenomenon, and therefore, multifactorial lifestyle interventions with sufficient support and follow-up strategies can be expected to be successful (47). Based on the results of this study, aiming to replace 1 h of SB with nonexercise PA is not effective in increasing insulin sensitivity in high-risk populations, even if it can help in weight control (7,24). However, according to the additional analyses, successfully reducing SB by ~30 min·d^−1^ mainly by increasing moderate-intensity PA might increase insulin sensitivity, but future studies are warranted to confirm this. Nevertheless, individual goals are clinically important, because what seems achievable to some might not be that for others. Therefore, sitting less can be a good starting point for individuals that find committing to increased PA unattainable.

## CONCLUSIONS

Reducing 40 min of daily SB mainly by adding nonexercise PA seems not to be enough to improve whole-body insulin sensitivity in adults with MetS in 6 months, although it minimally decreased fasting insulin. Instead, multifaceted approaches with sustained changes in SB and PA behaviors including exercise and a healthy diet are more likely to be beneficial in the long term.

## Supplementary Material

SUPPLEMENTARY MATERIAL
