# Supplementary material for: Reducing Sedentary Time and Whole-Body Insulin Sensitivity in Metabolic Syndrome: A 6-Month Randomized Controlled Trial
Source: Med Sci Sports Exerc. 2022 Oct 13;55(3):342–53. doi: 10.1249/MSS.0000000000003054 (PMC9924963; doi:10.1249/MSS.0000000000003054)
Supplement: SUPPLEMENTARY MATERIAL [file msse-55-342-s001.pdf]

REDUCING SEDENTARY TIME AND WHOLE-BODY INSULIN SENSITIVITY IN METABOLIC SYNDROME – A 6-MONTH RANDOMIZED CONTROLLED TRIAL

Tanja Sjöros[1], Saara Laine[1], Taru Garthwaite[1], Henri Vähä-Ypyä[2], Eliisa Löyttyniemi[3], Mikko Koivumäki[1], Noora Houttu[4], Kirsi Laitinen[4], Kari K Kalliokoski[1], Harri Sievänen[2], Tommi Vasankari[2,5], Juhani Knuuti[1], Ilkka HA Heinonen[1,6]

1: Turku PET Centre, University of Turku and Turku University Hospital, Turku, Finland

2: The UKK Institute for Health Promotion Research, Tampere, Finland

3: Department of Biostatistics, University of Turku, Turku, Finland

4: Institute of Biomedicine, University of Turku, Turku, Finland

5: Faculty of Medicine and Health Technology, Tampere University, Tampere, Finland

6: Rydberg Laboratory of Applied Sciences, University of Halmstad, Halmstad, Sweden

Supplementary material

Results

Supplementary figures.

Supplementary figure 1. Study flow diagram.

Study Flow Diagram

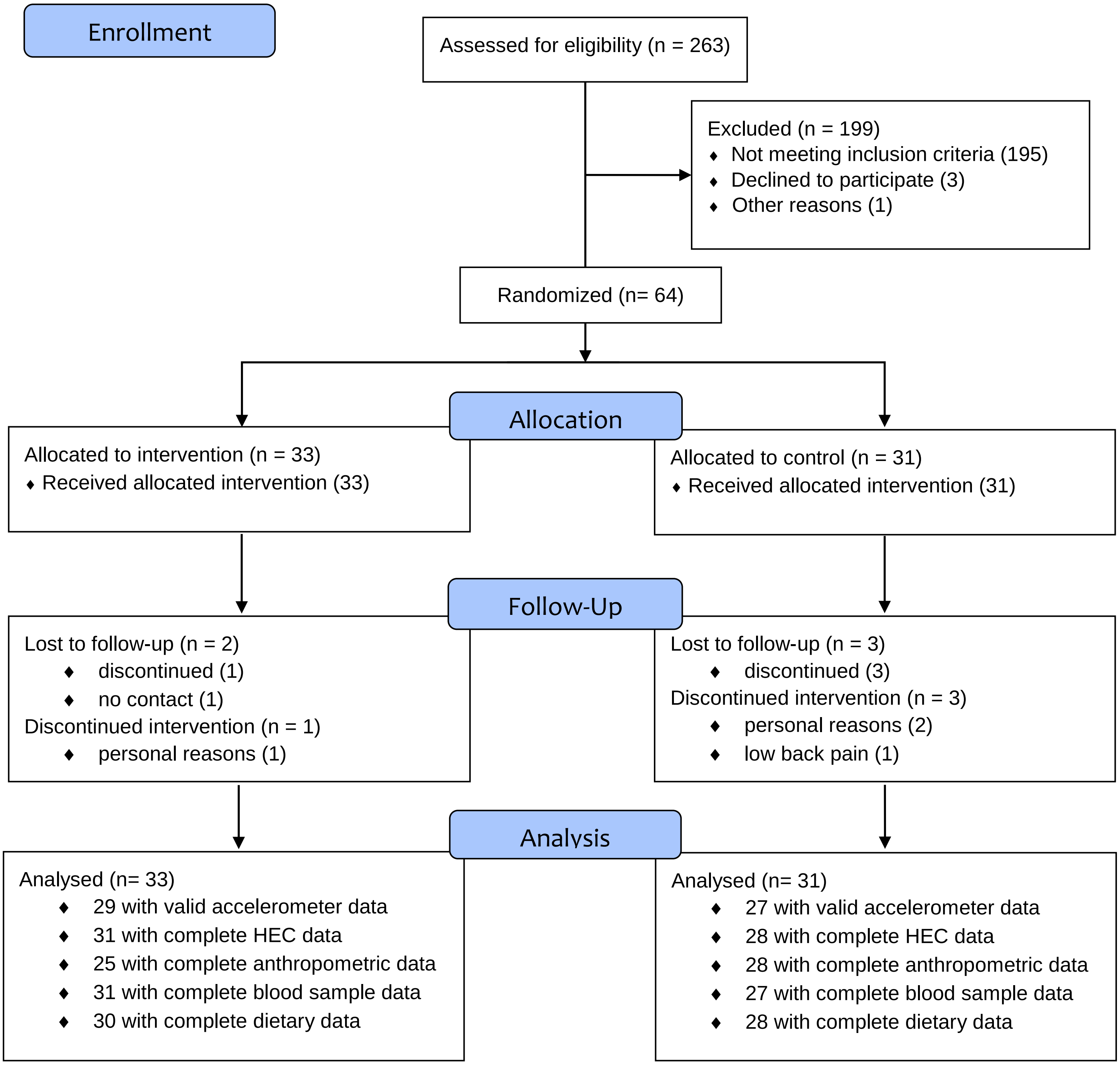

HEC, hyperinsulinemic euglycemic clamp.

Supplementary tables

Supplementary table 1. The plasma insulin and glucose values [mean (SD)] in the intervention and control groups during the hyperinsulinemic euglycemic clamp before and after the intervention.

	Pre		Post	
Group	Intervention	Control	Intervention	Control
HEC glucose, mmol/L	5.1 (0.3)	5.1 (0.3)	5.0 (0.3)	5.2 (0.3)
HEC insulin, mU/L	71.0 (13.2)	74.5 (14.4)	69.8 (14.9)	75.8 (14.8)

HEC, hyperinsulinemic euglycemic clamp.

Supplementary table 2. The numerical estimates [model-based LS means (SE)] of the results presented in the original figures 1, 3, 4, 5, and 6.

	INT		CONT	
	Screening	Intervention	Screening	Intervention
SB, h/day	10.2 (0.1)	9.5 (0.2)	10.2 (0.2)	10.1 (0.2)
Standing, h/day	1.8 (0.1)	1.9 (0.1)	1.8 (0.1)	1.7 (0.1)
LPA, h/day	1.7 (0.1)	1.9 (0.1)	1.8 (0.1)	1.9 (0.1)
MVPA, h/day	1 (0.1)	1.3 (0.1)	1 (0.1)	1.1 (0.1)
Steps, n/day	5326 (397)	8632 (408)	5150 (402)	6749 (426)
Breaks in SB, n/day	29 (2)	31 (2)	29 (2)	31 (2)
	Pre	Post	Pre	Post
BMI, kg/m^2	31.5 (0.8)	31 (0.8)	31.7 (0.8)	31.6 (0.8)
Body mass, kg (log10)	1.97 (0.01)	1.96 (0.01)	1.97 (0.01)	1.97 (0.01)
Waist circumference, cm	112 (1.9)	109.8 (1.9)	111.1 (1.9)	110 (1.9)
Body fat, %	42 (1.1)	41.3 (1.1)	42.6 (1.1)	41.7 (1.1)
MetS Score	0.96 (0.51)	0.29 (0.53)	-0.34 (0.53)	-0.45 (0.53)
Fat free mass, kg	54.4 (1.2)	54.1 (1.2)	54.1 (1.2)	54.7 (1.2)
M-value, µmol/kg/min (log10)	1.13 (0.05)	1.19 (0.05)	1.17 (0.05)	1.17 (0.05)

Fasting insulin, mU/L (log10)	1.03 (0.04)	0.98 (0.04)	1.02 (0.05)	1.06 (0.05)
Fasting glucose, mmol/L	5.9 (0.1)	5.9 (0.1)	5.8 (0.1)	5.8 (0.1)
HbA1c, mmol/mol	37.1 (0.5)	37.4 (0.5)	36.3 (0.5)	37.6 (0.5)
Energy intake, kJ/day	7359 (293)	7351 (303)	7829 (300)	7625 (310)
Energy intake, kJ/kg/day	79.4 (3.3)	80.4 (3.4)	84.7 (3.4)	82.3 (3.5)
	More Active		Continuously Sedentary	
	Pre	Post	Pre	Post
M-value, µmol/kg/min (log10)	1.12 (0.05)	1.2 (0.05)	1.18 (0.05)	1.16 (0.05)

INT, intervention group; CONT, control group; SB, sedentary behavior; LPA, light physical activity; MVPA moderate to vigorous physical activity; BMI, body mass index; MetS Score, sum score of (waist circumference, mean blood pressure, fasting plasma glucose, insulin, HDL/triglyceride-ratio); M-value, whole-body glucose uptake in hyperinsulinemic euglycemic clamp; HbA1c, glycated hemoglobin.
